# Predictors of postnatal depression in the slums Nairobi, Kenya: a cross-sectional study

**DOI:** 10.1186/s12888-022-03885-4

**Published:** 2022-04-05

**Authors:** Esther W. Kariuki, Mary W. Kuria, Fredrick N. Were, David M. Ndetei

**Affiliations:** Department of Psychiatry, School of Medicine, The University of Nairobi, P.O. Box 30197, GPO, Nairobi, Kenya

**Keywords:** Postnatal depression, Predictors, MCH, Low-resource settings, Slums

## Abstract

**Background:**

Postnatal depression (PND) is a universal mental health problem that prevents mothers’ optimal existence and mothering. Although research has shown high PND prevalence rates in Africa, including Kenya, little research has been conducted to determine the contributing factors, especially in low-resource communities.

**Objective:**

This study aimed to investigate the PND risk factors among mothers attending Lang’ata and Riruta Maternal and Child Health Clinics (MCH) in the slums, Nairobi.

**Methods:**

This study was cross-sectional. It is part of a large study that investigated the effectiveness of a brief psychoeducational intervention on PND. Postnatal mothers (567) of 6-10 weeks postanatal formed the study population. Depression rate was measured using the original 1961 Beck’s Depression Inventory (BDI). In addition, a sociodemographic questionnaire (SDQ) was used to collect hypothesized risk variables. Multivariable logistic regression analysis was used to explore predictors of PND.

**Results:**

The overall prevalence of PND in the sample of women was 27.1%. Women aged 18-24 (β = 2.04 95% C.I.[0.02; 4.05], *p* = 0.047), dissatisfied with body image (β = 4.33 95% C.I.[2.26; 6.41], *p* < 0.001), had an unplanned pregnancy (β = 2.31 95% C.I.[0.81; 3.80], *p* = 0.003 and felt fatigued (β = − 1.85 95% C.I.[− 3.50; 0.20], *p* = 0.028) had higher odds of developing PND. Participants who had no stressful life events had significantly lower depression scores as compared to those who had stressful life events (β = − 1.71 95% C.I.[− 3.30; − 0.11], *p* = 0.036) when depression was treated as a continuous outcome. Sensitivity analysis showed that mothers who had secondary and tertiary level of education had 51 and 73% had lower likelihood of having depression as compared to those with a primary level of education (A.O.R = 0.49 95% C.I.[0.31-0.78], *p* = 0.002) and (A.O.R = 0.27 95% C.I.[0.09-0.75], *p* = 0.013) respectively.

**Conclusion:**

This study reveals key predictors/risk factors for PND in low-income settings building upon the scanty data. Identifying risk factors for PND may help in devising focused preventive and treatment strategies.

## Background

Postnatal depression (PND) is a worldwide mental health problem [1] across countries and cultures [2]. The PND prevalence is higher (1.9% to 82.1%) in developing countries compared to (5.2% to 74.0%) in the developed countries [2]. In Africa, a systematic review and meta-analysis reported an overall pooled PND prevalence of 16.84% [3]. The few published research studies conducted in Kenya report high PND prevalence rates of 18.7% among mothers attending Mother and Child Health (MCH) clinics in urban, resource-poor environments [4], and 64.1% among mothers of children with severe acute malnutrition in general pediatric wards at Kenyatta national Hospitial [5].

Symptoms of PND include depressed mood, anxiety, anhedonia [6], fatigue [7], sleep difficulties [8] and concentration problems [8] which interfere with mothers’ parenting capacities [9]. The parenting incapacitation is associated with poor child’s physical health [10], and growth [11], and poor mother-child relationship [12]. These, in turn, lead to poor child’s developmental outcomes [13].

Postnatal depression is caused by many factors that may include: - psychosocial, socioeconomic, biological causes [14]. Some of the main psychosocial risk factors include; stressful life events [15], low income [16] and low education [17]. Besides, infant characteristics like adverse birth and infant health outcomes [3], difficult temperament [18], and unwanted gender [19] are risk factors. Mothers with poor physical health [20], poor obstetric histories [21], unplanned pregnancy [22], and a history of psychiatric illness [23] are likely to have PND. Moreover, poor environmental conditions [24] and cultural practices [25, 26] are also PND predictors.

Although PND is a public health problem, African countries [26], including Kenya, have neglected it considering the small number of published studies[18, 25]. Therefore, more research is needed in this area [3]. Moreover, it is crucial to investigate and understand PND risk factors in order to identify women at risk and offer them necessary intervention early enough [27]. This study aimed to investigate PND predictors in the early postnatal period (6-10 weeks) in two low-resourced urban communities. To the best of our knowledge, this is the first paper reporting prevalence and associated factors of depression postnatal mothers receiving MCH services in an urban slum setting in Kenya.

## Methods, design, participants and procedures

This was cross-sectional study conducted within a large study that investigated the effectiveness of a brief psychoeducational intervention on PND in the slums, Nairobi [28]. Data were collected from Lang’ata and Riruta Health Centres- MCH clinics. Both are situated in Nairobi County and serve low-resource communities.

The sampling method for the main study within which the PND work was conducted is published in an earlier paper [28]. Briefly, a calculated sample of 227 participants would detect a difference of 15% in PND reduction with 90% power at 5% level of significance. To allow for 10% attrition rate, a minimum of 250 mothers were recruited in each of the groups, giving a total of 500 postnatal mothers. All the mothers in the main study were included in this study.

Mothers were recruited as they brought their infants for the first MCH clinic visit. Mothers were informed about the nature of the study and their rights by a Community Health Nurse Research assistant. Those who agreed to participate voluntarily signed a written consent form. Then they filled up a self-administered Social-Demographic Questionnaire (SDQ) and the original (1961) Becks Depression Inventory (BDI) which was in English and Kiswahili version. The variables are listed in Table 1. Of the total 591 eligible mothers, 575 participated in the study, which made a response rate of 97.3%. Sixteen participants refused to participate in the study.

**Table 1:** Participants’ socio-demographic and health characteristics

Variable	Category	Frequency/mean (***N*** = 567)	Percentage (%)
Age in Years	18-24 Years	256	45.1
	25-30 Years	213	37.6
	31-40	98	17.3
Age years	Mean (sd), Range	25.9 (sd)	18-40
Education Level	Primary	207	36.5
	Secondary	303	53.4
	Graduate and above	57	10.1
Marital Status	Currently married	509	89.8
	Currently not Married	58	10.2
Employment Status	Employed	85	15
	Self-employed	102	18
	Unemployed	380	67
Monthly Income	< 10,000	198	34.9
	10,000-19,000	186	32.8
	20,000-29,000	103	18.2
	30,000 and Above	80	14.1
Suffering from Chronic Illness	No	538	94.9
	Yes	29	5.1
Satisfied with body image	No	74	13.1
	Yes	493	86.9
Conflict with any close relatives	No	501	88.4
	Yes	66	11.6
Have a Stressful life Event	No	406	71.6
	Yes	161	28.4
Pregnancy Planned	No	185	32.6
	Yes	382	67.4
Happy with baby’s Health	No	191	33.7
	Yes	376	66.3
Have Work Problems (fatigue)	No	432	76.2
	Yes	135	23.8
Age of the infant	6 Weeks	400	70.5
	7-10 Weeks	167	29.5
Gestation at birth	< 37 Weeks	53	9.3
	> = 37 Weeks	514	90.7

All participants were over 18 years of age and gave informed consent before they were enrolled in the study. We obtained ethical approval from Kenyatta National Hospital Ethical Committee (Ref. KNH-ERC/A/311- 13th July 2015), Office of the President through the Ministry of Higher Education Science and Technology and Chief Administrative Officer (CHS) Nairobi County. We confirm that all methods were performed in accordance with the Kenyatta National Hospital/University of Nairobi - Ethics & Research Committee guidelines and regulations.

## Data collection instruments

### Beck’s Depression Inventory (BDI)

The outcome of interest was depression, assessed using Beck’s Depression Inventory (BDI). It was created by Dr. Aaron T. Beck and first published in 1961 [29]. Beck’s Depression Inventory is a self-administered report which takes approximately 10 min to complete and has demonstrated internal consistency yielding a mean coefficient alpha of 0.86 for psychiatric patients and 0.81 for non-psychiatric persons [30]. It is also positively correlated with the Hamilton Depression Scale [30] with a Pearson correlation coefficient of 0.71. The test was also found to have a high one-week test-retest reliability with a Pearson value of 0.93 [31]. BDI comprises 21 groups of statements describing the way one has been feeling during the past two weeks, including today. The response options range from 0 to 3, a value assigned to each answer to give the total score compared to a key to determine the severity of depression. A total score is calculated as the sum of the 21 items with a range of 0-63. The clinical cut-offs are 11-16 (mild mood disturbance), 17-20 (borderline clinical depression), 21-30 (moderate depression), 31-40 (severe depression), and 40-63 (extreme depression). The BDI has been successfully used in Kenya and other countries [32–34].

## Sociodemographic questionnaire

A sociodemographic questionnaire was used to collect personal information and hypothesized PND risk factors that include: - mother’s age; educational level; marital status; monthly household income; suffering from chronic illness; satisfaction with body image; conflict with any close relatives; have a stressful life event; pregnancy planned; happy with infants’ health; not able to work (fatigue) and age of the infant and gestational age. The variables are listed in Table 1.

## Data analysis

Item means, standard deviations, frequencies, and percentages were calculated for the sociodemographic, psychosocial related variables as well as for the depression scores. Crude association between each independent variable and the dependent variable (PND) was assessed using bivariate analyses. Variables with *p*-value < 0.25 for the crude association were entered into a multivariable generalized linear regression model to control the cofounders and identify independent predictors of PND. The *p*-value of < 0.25 was used in order not omit variables that might have an influence to the outcome variables but have been confounded by other variables. *P*-value of < 0.05 was used as the criterion for statistical significance, and beta coefficients with a 95% confidence interval was used to indicate the strength of association. Sensitivity analysis was conducted using multivariable logistic regression between those with probable clinical depression (EPDS scores of ≥21) and those without depression ((EPDS scores of < 21). All analyses were conducted with IBM SPPS v 23.

## Results

### Sociodemographic and other characteristics of the participants

Table 1 reports the characteristics of the participants in our sample. The mean age was 25.9 years and ranged from 18 to 40. The majority of the participants (45.1%) were aged between 18 and 24 years; 37.6% were aged between 25 and 30 years; 17.3% were aged between 30 and 40 years. The majority (89.8%) were married, more than half (53.4%) had secondary level of education, nearly 36.5% had a primary education level, the rest, (10.1%) had tertiary level of education. The majority (67.7%) of the participants were earning an income below 20,000 Kenya Shillings (about 196$) per month. 5.1% of the women had been suffering from chronic illness, 86.9% were satisfied with their body image, 11.6% had a conflict with their relatives, 28.4% had a stressful life event, 67.4% had planned their pregnancy, 66.7% were happy with the current baby’s health, and 23.8% had work-related problems (fatigue). The majority (70. 5%) had infants aged 7-10 weeks, while the rest had infants aged 6 weeks. About 9.3% had babies born before 37 weeks (pre-term births).

### Prevalence of postnatal depression

**Fig. 1:**
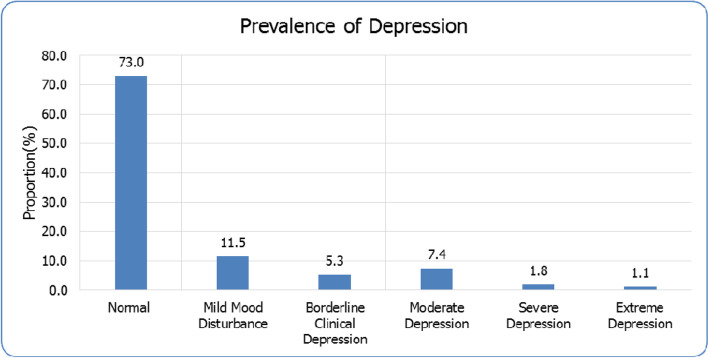
Prevalence of depression

As presented in Fig. 1, PND scores based on the original (1961) Becks Depression Index (BDI0 cut-off points, were as follows: Normal (0-10), (*n* = 414, 73.0%); Mild mood disturbance (11-16); (*n* = 65, 11.5%); Borderline clinical depression (17-20); (*n* = 30, 5.3%); Moderate depression (21-30); (*n* = 42, 7.4%); Severe depression (31-40); (*n* = 10, 1.8%) and Extreme depression (40+); (*n =* 6, 1.1%). Therefore, the PND prevalence is 27.0%. The mean BDI score was 7.73, SD = 8.9 and range was 0-54.

### Independent predictors of postnatal depression

As is shown in Table 2, after adjusting for all factors that were associated with postnatal depression at the bivariate level (*p* < 0.25) using generalized linear models, mothers who were younger (18-24 years) had significantly higher depression scores as compared to those who were older (31-40 years) (β = 2.04 95% C.I.[0.02; 4.05], *p* = 0.047). Mothers who were unsatisfied with their body image had significantly higher depression scores as those who were satisfied with their body image (β = 4.33 95% C.I.[2.26; 6.41], *p* < 0.001). Mothers who had no stressful life events had significantly lower depression scores compared to those who had stressful life events (β = − 1.71 95% C.I.[− 3.30; − 0.11], *p* = 0.036). Mothers who had an unplanned pregnancy had significantly higher depression scores than those who had a planned pregnancy (β = 2.31 95% C.I.[0.81; 3.80], *p* = 0.003). Mothers who did not report physical exhaustion (fatigue) had significantly lower depression scores as compared to those who did (β = − 1.85 95% C.I.[− 3.50; 0.20], *p* = 0.028).

**Table 2:** Independent predictors of depression

Variable	Category	Multivariable Analysis		
		β(s.e)	95% C.I	*p*-value
Age in Years	18-24	2.04(1.03)	[0.02; 4.05]	**0.047**
	25-30	1.11(1.05)	[−0.94; 3.17]	0.289
	31-40	Ref.		
Education Level	Primary	2.56(1.36)	[−0.10; 5.22]	0.060
	Secondary	−0.09(1.28)	[−2.60; 2.41]	0.941
	Graduate and above	Ref.		
Employment Status	Employed	−1.56(1.08)	[−3.67; 0.55]	0.148
	Self-employed	− 1.26(0.97)	[− 3.16; 0.64]	0.193
	Unemployed	Ref.		
Monthly Income	< 10,000	1.43(1.18)	[−0.88; 3.74]	0.225
	10,000-19,000	0.60(1.19)	[−1.73; 2.92]	0.614
	20,000-29,000	0.09(1.29)	[−2.45; 2.63]	0.944
	30,000 and above	Ref.		
Satisfied with body image	No	4.33(1.06)	[2.26; 6.41]	**< 0.001**
	Yes	Ref.		
Conflict with any close relatives	No	−1.95(1.12)	[−4.15; 0.25]	0.082
	Yes	Ref.		
Have a Stressful life Event	No	−1.71(0.81)	[−3.30; −0.11]	**0.036**
	Yes	Ref.		
Pregnancy Planned	No	2.31(0.76)	[0.81; 3.80]	**0.003**
	Yes	Ref.		
Have Work Problems	No	−1.85(0.84)	[−3.50; −0.20]	**0.028**
	Yes	Ref.		

### Independent predictors of depression (sensitivity analysis results)

As shown in Table 3, after adjusting for all factors that were associated with postnatal depression at the bivariate level (*p* < 0.25) using generalized linear models. Mothers who had secondary and tertiary level of education had 51 and 73% lower likelihood of having depression as compared to those with primary level of education (A.O.R = 0.49 95% C.I.[0.31-0.78], *p* = 0.002) and (A.O.R = 0.27 95% C.I.[0.09-0.75], *p* = 0.013) respectively. Mothers who were satisfied with their body image had 63% lower likelihood of having depression as compared to those who were not (A.O.R = 0.37 95% C.I.[0.21-0.67], *p* = 0.001). Mothers who had conflicts with relatives were 2.37 times more likely to have depression as compared to those who don’t (A.O.R = 2.37 95% C.I.[1.28-4.28], *p* = 0.006). Mothers who had planned pregnancy had 50% lower likelihood of having depression as compared to those who had unplanned pregnancy (A.O.R = 0.50 95% C.I.[0.32-0.79], *p* = 0.003). Mothers who had work related problems were 2.11 times more likely to have depression as compared to those who didn’t (A.O.R = 2.11 95% C.I.[1.29-3.47], *p =* 0.003).

**Table 3:** Independent Predictors of depression BDI (Sensitivity Analysis)

Variable	Category	A.O.R.(95% C.I)	***P***-value
Age in Years	18-24 Years	1	
	25-30 Years	0.68(0.41-1.14)	0.144
	31-40	0.68(0.36-1.29)	0.243
Education Level	Primary	1	
	Secondary	0.49(0.31-0.78)	**0.002**
	Graduate and above	0.27(0.09-0.75)	**0.013**
Employment Status	Employed	1	
	Self-employed	0.82(0.32-2.08)	0.676
	Unemployed	1.55(0.73-3.26)	0.252
Monthly Income	< 10,000	1	
	10,000-19,000	0.89(0.53-1.50)	0.672
	20,000-29,000	0.54(0.26-1.12)	0.097
	30,000 and Above	0.95(0.44-2.05)	0.900
Satisfied with body image	No	1	
	Yes	0.37(0.21-0.67)	**0.001**
Conflict with any close relatives	No	1	
	Yes	2.33(1.28-4.26)	**0.006**
Have a Stressful life Event	No	1	
	Yes	1.22(0.75-1.97)	0.423
Pregnancy Planned	No	1	
	Yes	0.50(0.32-0.79)	**0.003**
Have Work Problems	No	1	
	Yes	2.11(1.29-3.47)	**0.003**

## Discussion

The prevalence of PND was 27%. Published literature from Kenya shows high PND prevalence rates in different settings: 18.7% among women attending MCH clinics in two public hospitals in Nairobi [4] and 64.4% among mothers whose children were admitted to hospital due to severe acute malnutrition at Kenyatta National Hospital. The 27% PND prevalence rate is relatively lower than the reported 64.4% among women with babies with severe acute malnutrition [5], and higher than the 18.7% in mothers attending public hospitals. This indicate that the PND prevalence rate in this study is comparable but slightly higher than that of women attending the public hospitals which is expected since the mothers in this sample were exclusively from the slum settings and therefore extreme poverty. Recent research findings from other African countries show a high PND prevalence rates: Rwanda 63.6%, South Africa (57.14%) [35] and (38.8%) [36], Nigeria 35.6% [37]. Comparable to our finding, a systematic review and meta-analysis revealed a prevalence rate of 26% in Middle-East countries, while European countries had lower rates (8%) [38].

Regarding maternal age, younger mothers (18-24 years) had significantly higher depression scores than older (31-40 years) mothers. This is corroborated by other research findings [39–42]. The young mothers are usually inexperienced in taking up the challenging new maternal role [43], and therefore prone to maternal distress in several aspects including stress, adaptation, functioning and connecting [44]. Conversely, Smorti et al.’s study showed that older mothers are more likely to develop PND compared to young ones [27]. However, many other research studies have not found any association between maternal age and PND [45–47].

Women dissatisfied with their body image had a significant effect on the risk for developing PND symptoms in this study. Similar findings have been reported by Riquin et al. [48] and Hartley et al. [49]. Whereas most pregnancy and childbirth is naturally associated with weight gain due to the physiological changes involved, women are confronted with social pressures to maintain a pre-pregnant small size and shaped body in the postnatal period, contributing to their self-image perception [50, 51]. On the other hand, better body image is highly protective of developing PND [52]. In contrast, another research study showed that body image dissatisfaction was consistent but weakly associated with PND [53].

Participants who had no stressful life events had significantly lower depression scores than those who had stressful life events in this study. This suggests that negative life events are predictive of PND [54–56].

This study replicates the finding that unplanned pregnancy is a risk factor for PND, as other studies have shown [57, 58]. It is possible that the circumstances surrounding the unplanned pregnancy and the consequences resulting from having the pregnancy altogether amount to a stressful experience. For example, unplanned pregnancy has negative consequences that include; stigma, perceived loss of opportunities [59], poor health [60] and unhappiness [61].

Women who felt fatigued and unable to perform the usual household chores after giving birth were more depressed than those who did not. Correlating with our findings, several research studies have shown that that mothers in the high-risk depressive symptoms group were most likely to complain of fatigue [62–64]. It should be noted that depressive symptoms and physical fatigue overlap but have distinct trajectories [65, 66] and should be best understood as separate psychological constructs or experiences [67]. Research studies have recommended differentiating fatigue and depression for postnatal mothers [66, 68] to improve early postnatal care [65], which is a limitation in this study.

Sensitivity analysis was consistent with the main analysis, apart from mothers with secondary and tertiary levels of education, who were found to have a lower likelihood of having PND than those with a primary level of education. Therefore, this study suggests that high education may be a protective factor for PND, as other studies have shown [69, 70]. But Miyake et al. study found no association between maternal education and PND [71].

## Strengths and limitations

### Strengths

- To the best of our knowledge, this is the first paper reporting prevalence and associated factors of postnatal depression among mothers seeking services in an urban slum setting.
- Depression was measured through face-to-face interviews using the original BDI questionnaire.

### Limitations

- Data on sociodemographic and lifestyle factors were collected using self-reports, which is prone to reporting bias.
- In this study, fatigue was associated with PND although it can be understood as a separate construct.

This cross-sectional study cannot establish causal associations between postnatal depression and the risk factors examined. The generalizability of our findings is limited to settings that are similar to our study population. Future studies including larger and diverse populations are recommended.

## Conclusion

This study builds upon the scant previous studies on PND from low-income countries. Identifying mothers at risk will lead to timely intervention, preventing PND and its progression.

## Data Availability

The availability of data for this manuscript is available upon formal request to the corresponding author.

## Abbreviations

PND: Postnatal Depression
MCH: Maternal and Child Health
SDQ: Sociodemographic Questionnaire
BDI: Beck’s Depression Inventory
CHS: Chief Administrative Officer
